# Simvastatin Improves Cardiac Function through Notch 1 Activation in BALB/c Mice with Chronic Chagas Cardiomyopathy

**DOI:** 10.1128/AAC.02141-19

**Published:** 2020-07-22

**Authors:** Daniela Guzmán-Rivera, Ana Liempi, Fabiola González-Herrera, Sebastián Fuentes-Retamal, Ileana Carrillo, Patricio Abarca, Christian Castillo, Ulrike Kemmerling, Barbara Pesce, Juan Diego Maya

**Affiliations:** a Clinical and Molecular Pharmacology Program, Institute of Biomedical Sciences, Faculty of Medicine, University of Chile, Santiago, Chile; b Anatomy and Developmental Biology Program, Institute of Biomedical Sciences, University of Chile, Santiago, Chile

**Keywords:** Chagas cardiomyopathy, Notch 1, simvastatin

## Abstract

Chagas disease, caused by the protozoan Trypanosoma cruzi, endemic in Latin America but distributed worldwide because of migration. Without appropriate treatment, the disease progresses from an acute asymptomatic phase to a chronic, progressive inflammatory cardiomyopathy causing heart failure and death. Despite specific trypanocidal therapy, heart damage progression cannot be stopped or reversed. Statins, as part of their pleiotropic actions, can modulate chagasic myocarditis by inducing the production of 15-epi-lipoxin A_4_ (15-epi-LXA_4_), a proresolution lipid mediator in inflammation. Furthermore, several reports suggest that simvastatin activates the Notch pathway after stroke in cerebral endothelial cells, enhancing blood flow by promoting angiogenesis. Thus, statins are an attractive therapeutic strategy for modulating the Notch pathway to reverse the chronic heart damage induced by T. cruzi. BALB/c mice chronically infected with T. cruzi were treated with 1 mg/kg/day simvastatin or 25 μg/kg/day 15-epi-LXA_4_ for 20 days. During the treatment period, cardiac function was evaluated by echocardiography. At 80 days postinfection, the heart tissues were assessed for Notch 1 activity. T. cruzi infection activated the Notch 1 pathway, and simvastatin (but not 15-epi-lipoxin A_4_) produced a further increase in that activity, correlating with improvement in the ejection fraction and histopathologic findings typical of T. cruzi infection, including improvements in inflammation and fibrosis. Moreover, simvastatin increased the number of isolectin B4-positive cells, suggesting active angiogenesis in the chronically infected hearts without alteration of the parasitic load. Simvastatin, probably acting through the Notch 1 pathway, decreases inflammation, improving cardiac function in mice chronically infected with T. cruzi.

## Abstract (precis)

Chagas disease, caused by the protozoan Trypanosoma cruzi, endemic in Latin America but distributed worldwide because of migration. Without appropriate treatment, the disease progresses from an acute asymptomatic phase to a chronic, progressive inflammatory cardiomyopathy causing heart failure and death. Despite specific trypanocidal therapy, heart damage progression cannot be stopped or reversed. Statins, as part of their pleiotropic actions, can modulate chagasic myocarditis by inducing the production of 15-epi-lipoxin A_4_ (15-epi-LXA_4_), a proresolution lipid mediator in inflammation.

## INTRODUCTION

Notch signaling crucially regulates heart and vessel development during embryogenesis. Thus, mutations in proteins of this pathway result in congenital malformations (1). Notch is a highly conserved signaling pathway regulating cell differentiation during development and adulthood (2). The single-pass transmembrane Notch receptor contains an intracellular domain with transcriptional regulatory activity after cleavage from the membrane by a γ-secretase following ligand-receptor interactions due to cell-to-cell contact. The Notch ligands belong to the delta-like (Dll1 to Dll4) or jagged (Jag1 and Jag2) families. The N-terminal intracellular domain (NICD) arises from the cleavage at the Gly^1743^ and Val^1744^ residues within the transmembrane domain (the S3 site) in the receptor cell (3). Then, NICD is translocated to the nucleus, where it interacts with the CSL (CBF1, Suppressor of Hairless, Lag-1) DNA-binding protein, acting to release corepressors of Notch target genes and allowing interaction of the MAML1 (mastermind-like transcriptional coactivator 1) protein with DNA (4). Notch-activated genes include repressors such as the Hairy and Enhancer of Split (Hes1) and Hairy/Enhancer of split-related with YRPW motif (HEY1), which are involved in a negative-feedback loop that controls the expression of the proinflammatory cytokines interleukin-6 (IL-6) and IL-12 and Hes and Hey family proteins (5), as well as other tissue-specific proteins.

In adulthood, the Notch pathway is reactivated after cardiac ischemia or cerebral stroke to decrease the ischemic area, inducing angiogenesis in endothelial cells or Notch-activated stem cells (6–8), a fact corroborated by the detection of NICD overexpression after myocardial infarction (MI) in mice (9). In the context of myocardial ischemia, a reduction in fibrosis and improved cardiac function related to activation of the Notch pathway are observed (10). Moreover, the administration of the Jag1 ligand improved cardiac fibrosis in a rat model of MI (11).

Chronic Chagas heart disease, caused by the hemoflagellate protozoan Trypanosoma cruzi, is the ultimate complication, presenting with decreased cardiomyocytes and fibrosis as a product of a chronic inflammatory process, leading to heart failure, arrhythmias, and death of affected individuals (12). Previously, we reported that simvastatin decreases T. cruzi-elicited heart inflammation and endothelial activation by increasing the production of the proresolving lipid 15-epi-lipoxin A_4_ (15-epi-LXA_4_) (13). Furthermore, it was reported that cholesterol-lowering statins can modulate Notch 1 activity, increasing angiogenesis in ischemic brains of rats (14), probably by increasing presenilin (Psn1) activity (15), a component of γ-secretase.

Considering that current specific trypanocidal therapy is less than 100% effective, drugs that modify host factors may be an alternate way to treat this disease. Thus, the present work aims to study the effect of simvastatin on the activity of the Notch signaling pathway, the role of 15-epi-LXA_4_ as a mediator of this activity, and the impact of this treatment on cardiac function and angiogenesis in a murine model of chronic T. cruzi heart infection.

## RESULTS

### Simvastatin activates the Notch 1 pathway in the hearts of T. cruzi-infected mice.

**FIG 1:**
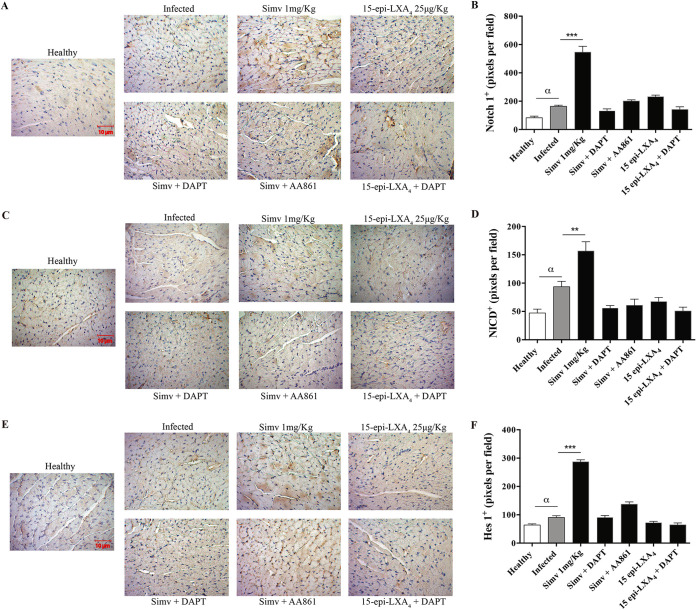
Simvastatin (Simv) increases the expression and activation of the Notch receptor and signaling pathway in chronically T. cruzi-infected mice. BALB/c mice were infected with T. cruzi trypomastigotes and treated with simvastatin on day 60 postinfection for 20 days. (A, C, and E) Representative Notch 1 receptor, NICD, and Hes1 immunohistochemistry in cardiac tissues. (B, D, and F) Semiquantitative analysis of the immunohistochemistry signal obtained from cardiac tissues for Notch 1 receptor, NICD, and HES1, respectively. Data from one experiment with five animals per group are expressed as the number of pixels ± standard errors. One-way ANOVA with Dunnett’s posttest was used to determine significance. **, *P ≤ *0.01; ***, *P ≤ *0.001, for results compared with those of infected mice; α, *P ≤ *0.05, for results comparing with healthy mice.

Figure 1 shows that the Notch 1 receptor, the NICD fragment, and Hes1 protein are present in BALB/c mice, indicating that this pathway is present in the heart tissue and is functional at 80 days postinfection (dpi) with T. cruzi. Moreover, the parasite induced a slight but significant increase in the immunostaining for Notch 1 (Fig. 1A and B) and the activity of the Notch pathway, as assessed by NICD (Fig. 1C and D) and Hes1 (Fig. 1E and F) immunostaining compared with levels in healthy mice. However, the most important finding is further activation of the pathway with simvastatin, including a significant increase in Notch 1 receptor immunostaining (*P < *0.001), a response blunted by inhibition of γ-secretase. Contrary to expectations, 15-epi-LXA_4_ did not modify Notch 1 activity or receptor expression.

**FIG 2:**
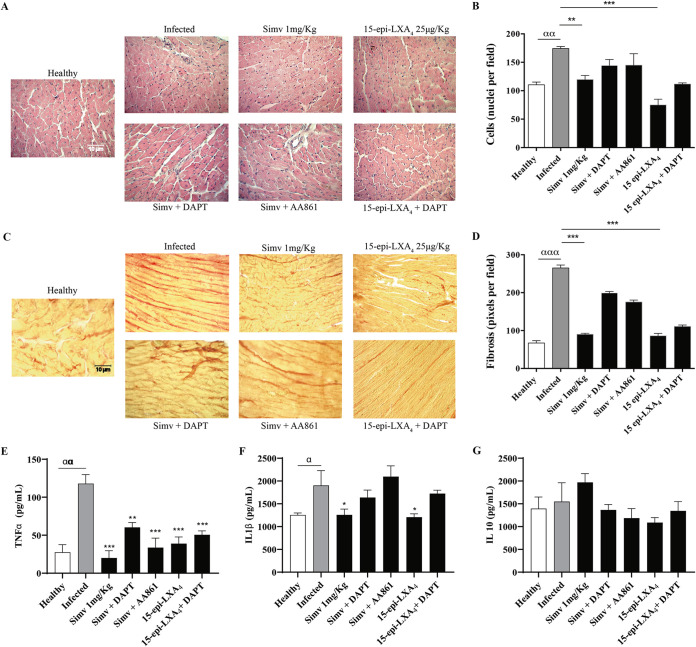
Simvastatin and 15-epi-lipoxin A_4_ decrease cardiac tissue damage and fibrosis in chronically T. cruzi-infected BALB/c mice. Mice were infected with T. cruzi trypomastigotes and treated with simvastatin on day 60 postinfection for 20 days. (A) Images of hematoxylin-eosin staining of representative cardiac tissues. (B) Semiquantitative analysis of cellularity in the cardiac tissues from five fields per animal. (C) Images of Picrosirius red staining of representative cardiac tissues. (D) Semiquantitative analysis of the red pixels in cardiac tissue, corresponding to the fibrosis indicator, of five fields per animal. (E to G) Concentration of TNF-α, IL-1β, and IL-10, as indicated, in the serum of the infected BALB/c mice. Determinations were performed by ELISAs. Data from one experiment with five animals are expressed as the number of pixels or nuclei ± standard errors. One-way ANOVA with Dunnett’s posttest was used to determine significance. ***, *P ≤ *0.001; **, *P ≤ *0.01; *, *P* ≤ 0.05, for results compared to those with infected mice; ααα, ≤ 0.001; αα, *P* ≤ 0.01; α, *P* < 0.05, for results compared to those with healthy mice.

To determine the role of simvastatin-modified Notch in chronic Chagas cardiomyopathy, we evaluated cellular infiltrate and fibrosis. As shown in Fig. 2A, on day 80 postinfection, disorganization of the myocardial architecture, in addition to an intense inflammatory infiltrate, induced by the parasite was observed. Simvastatin and 15-epi-LXA_4_ decreased the infiltrate. This response was blunted by the inhibition of 15-epi-LXA_4_ production with the 5-lipoxygenase (5-LOX) inhibitor AA861. Similarly, DAPT (*N*‐[*N*‐(3,5‐difluorophenacetyl)‐l‐alanyl]‐*S*‐phenylglycine *t*‐butyl ester) prevented the clearance of the inflammatory infiltrate although the association between DAPT and simvastatin or 15-epi-LXA_4_ was not clear because, in both cases, the anti-inflammatory effect of the statin predominated (Fig. 2B). Furthermore, simvastatin (and 15-epi-LXA_4_) prevented the increase in collagen deposition in cardiac tissue induced by the presence of T. cruzi (Fig. 2C and D), an effect reversed by the corresponding Notch 1 and 5-LOX inhibitors.

Tumor necrosis factor alpha (TNF-α) and IL-1β levels were increased by T. cruzi infection, but the level of IL-10 did not change in this setting. Simvastatin decreased inflammatory cytokines without changing IL-10 levels. However, the combination of simvastatin with DAPT produced a trend toward increasing levels of TNF-α and IL-1β without affecting IL-10 although these cytokines never reached the levels observed with the infection. Similarly, 15-epi-LXA_4_ decreased TNF-α and IL-1β levels, and 5-LOX inhibition partially reversed the effect of simvastatin on TNF-α and IL-1β.

**FIG 3:**
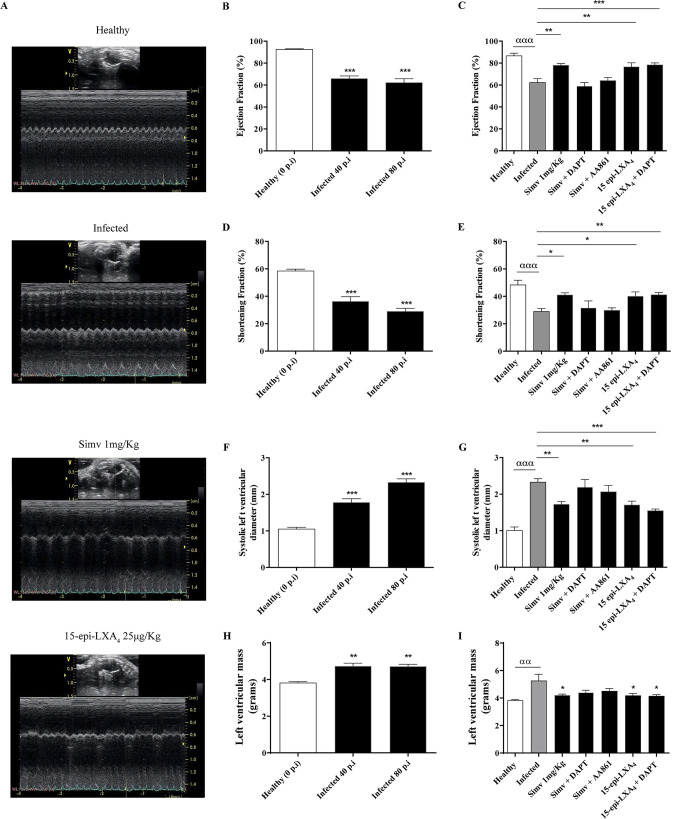
Simvastatin normalizes cardiac function in chronically T. cruzi-infected BALB/c mice. The mice were lightly sedated, and bidimensional echocardiography was performed with a Vivid-i instrument, using an iRL-RS 5- to 13-MHz transducer. (A) Representative bidimensional echocardiograms on day 80 postinfection. (B) Ejection fraction analysis at 0, 40, and 80 days postinfection. (C) Ejection fraction analysis at 80 dpi with the treatments labeled in the graph. (D) Fractional shortening at 0, 40, and 80 dpi. (E) Fractional shortening at 80 dpi with the indicated treatments. (F) Systolic left ventricular diameter at 0, 40, and 80 dpi. (G) Systolic left ventricular diameter at 80 dpi with the indicated treatments. (H) Left ventricular mass at 0, 40, and 80 dpi. (I) Left ventricular mass at 80 dpi with the indicated treatments. Data correspond to the means ± standard errors from one experiment (*n* = 9). One-way ANOVA with Dunnett’s posttest was used to determine significance. ***, *P* ≤ 0.001; **, *P* ≤ 0.01; *, *P* ≤ 0.05, for results compared with those of the infected control; ααα, *P* ≤ 0.001; αα, *P* ≤ 0.01, for results compared with those of healthy controls.

To evaluate the impact of simvastatin treatment, we obtained bidimensional echocardiograms at days 40 and 80 postinfection (Fig. 3A). In the infected mice, the ejection fractions (EF) at days 40 and 80 postinfection showed a significant decrease (Fig. 3B). As shown in Fig. 3C, simvastatin caused a significant but slight increase in EF (from 66.1% ± 2.32% to 78.0% ± 1.58%; *P* = 0.0024). This improvement in EF was also observed with 15-epi-LXA_4_ (*P* = 0.0037), correlating with the decrease in fibrosis. When Psn1 activity was inhibited, the simvastatin-induced increase in EF was not observed, suggesting the participation of Notch 1 in the statin-mediated improvement of this parameter. DAPT did not affect the effect of 15-epi-LXA_4_ on EF. Consistent with the decrease in EF, the fractional shortening decreased in the infected mice (Fig. 3D). There was a partial recovery of the fractional shortening with simvastatin (Fig. 3E). Additionally, 15-epi-LXA_4_ partially improved the fractional shortening, but this effect was not altered by DAPT (Fig. 3E). The diameter of the left ventricle (LV) in systole increased with T. cruzi infection at day 80 postinfection (Fig. 3F), which was improved by simvastatin (Fig. 3G), consistent with the improvement in ventricular function. The increase observed in the LV mass during infection, indicative of hypertrophy (Fig. 3H), decreased with simvastatin. For all parameters determined, DAPT dampened the effects of simvastatin; however, the beneficial action of 15-epi-LXA_4_ was not reversed by DAPT.

**FIG 4:**
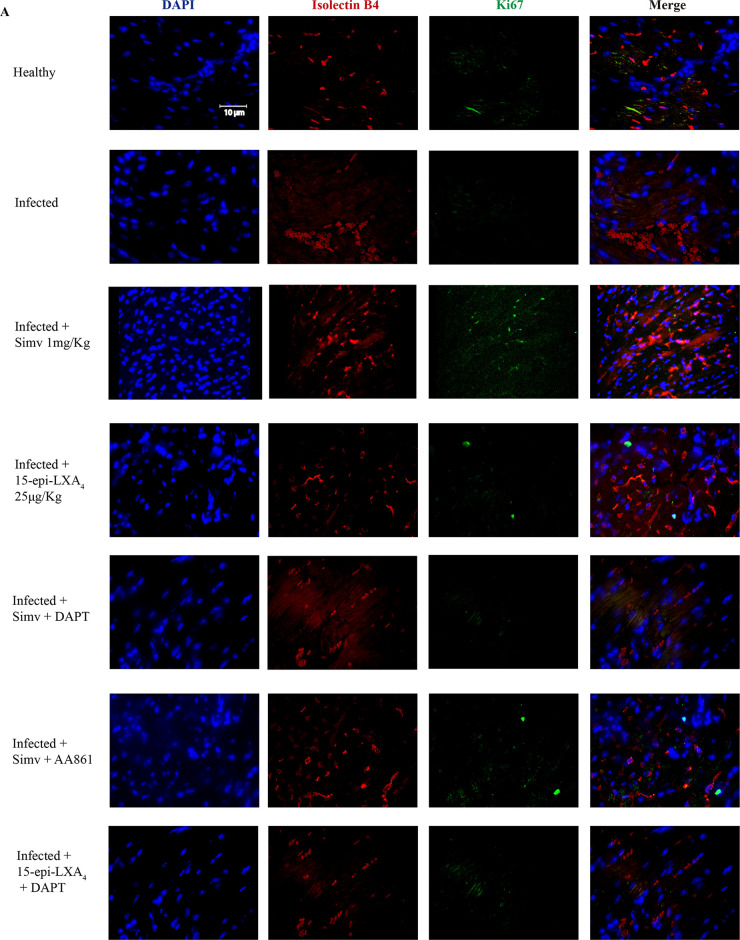

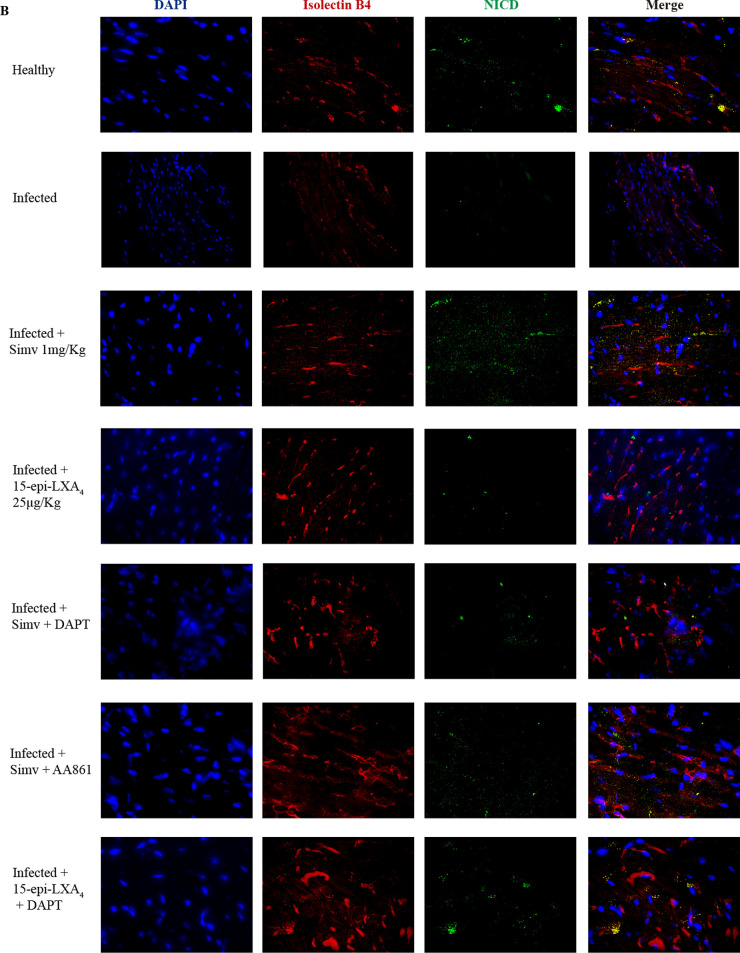
Simvastatin promotes angiogenesis in chronically T. cruzi-infected BALB/c mice. Mice were infected with T. cruzi trypomastigotes and treated with simvastatin on day 60 postinfection for 20 days. (A) Images of Ki67 and isolectin B4 staining of representative cardiac tissues. (B) Images of NICD and isolectin B4 staining of representative cardiac tissues. Images from one experiment with five animals per condition are shown.

Figure 4A shows the localization of the cell proliferation signal, as indicated by Ki67 and isolectin B4 staining of cells in the heart tissues of the mice treated with simvastatin. This type of signal was not observed when the mice were treated with 15-epi-lipoxin A_4_, which is consistent with other results described above. Notably, isolectin B4-positive (isolectin B4^+^) cell density decreased in the infected tissue. Complementarily, in Fig. 4B, the NICD signal was observed when the mice were treated with simvastatin, predominantly colocalizing with isolectin B4^+^ cells. Although some signals were observed in the tissues of the mice to which the Psn1 inhibitor was administered, the signals induced by simvastatin were stronger. These results were not the same as those with 15-epi-LXA_4_ or with the inhibitor of its synthesis.

**FIG 5:**
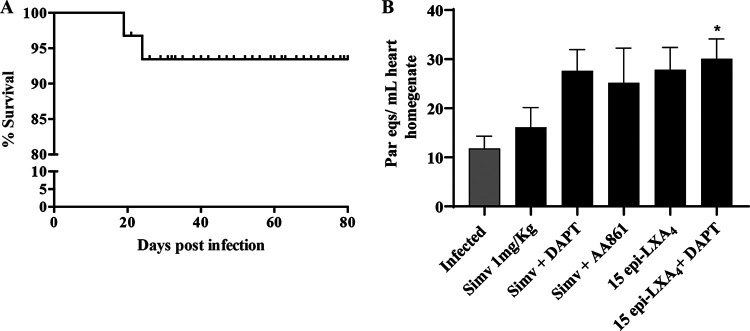
Survival analysis and cardiac parasite load in chronically T. cruzi-infected BALB/c mice. Mice were infected with T. cruzi trypomastigotes and treated with simvastatin on day 60 postinfection for 20 days. (A) Kaplan-Meier survival analysis from one experiment (*n* = 9). (B) Parasite load as determined by qPCR from the hearts of mice on day 80 postinfection. Results are expressed as the number of parasite equivalents per milliliter of heart homogenate. Data are expressed as the means ± standard errors from one experiment (*n* = 9). One-way ANOVA was performed to identify significant differences. *, *P* ≤ 0.05.

Remarkably, the survival of the mice was higher than 95% throughout the experiment, and there was no effect on animal mortality by the treatments used (Fig. 5A). Additionally, drug treatments did not significantly alter the T. cruzi DNA content in the cardiac tissue (Fig. 5B), ruling out any direct trypanocidal impact of the treatments on the parasite.

## DISCUSSION

Chronic Chagas disease continues to be a therapeutic challenge since specific antiparasitic treatment is not enough to prevent, in certain cases, the cardiac structural alterations that lead to chronic cardiomyopathy, which has high morbidity and mortality rates. Thus, pharmacological alternatives that intervene in host-related aspects, contributing to the overall improvement of the patient, should be explored.

Simvastatin, due to its anti-inflammatory effect, may contribute to the reduction of microvascular alterations induced by the persistence of T. cruzi in cardiac tissue, improving endothelial function throughout a 15-epi-LXA_4_-mediated effect (16). However, the Notch 1 pathway, which participates in cardiac angiogenesis, can be activated by simvastatin, as has already been reported in animal models of cerebral ischemia (14). The effect of simvastatin on Notch 1 signaling and its relationship with cardiac function have not been evaluated previously in T. cruzi-infected hearts.

In adult BALB/c mice, Notch pathway components are present in normal heart tissue. Furthermore, in the present murine model of chronic Chagas disease, the Notch pathway is activated, which is consistent with previous results in adipose tissue in the context of general inflammation induced by T. cruzi (17). This finding is logical because as part of the complex process of activation of the inflammatory response, cytokines such as TNF-α could be influenced by Notch pathway activity (18). Conversely, TNF-α can regulate Notch signaling (19), explaining why there was an increase in Notch, as evidenced by the immunohistochemistry analysis.

T. cruzi infection induced chronic heart inflammation, demonstrated by a significant increase in cell infiltration, fibrosis, and production of the proinflammatory cytokines TNF-α and IL-1β. Therefore, this process altered cardiac function, as evidenced by a decrease in the ejection fraction and significant structural changes, such as hypertrophy (increase in the mass of the LV) and dilatation (LV diameter increase).

Previously, we described 15-epi-LXA_4_ as a simvastatin intermediate that could decrease endothelial activation during chronic heart disease (13, 16). Our results corroborate these findings as inhibition of 5-LOX, which decreased 15-epi-LXA_4_, reversed the effects of simvastatin, increased cell infiltration or cardiac fibrosis, as well as the production of TNF-α and IL-1β, and decreased cardiac function. These findings are consistent with a chronic model of Chagas disease in dogs, where simvastatin also significantly decreased inflammation and cardiac function (20). The proresolution arachidonic acid-derived lipids lipoxins A_4_ and B_4_ are the products of 5-lipoxygenase (5-LOX) (21). Cyclooxygenase 2, when covalently modified by aspirin (acetylated) or by statins (nitrosylated), switches to 15-LOX activity, generating a derivative that will be further converted by 5-LOX to the epimer of LXA_4_, 15-epi-LXA_4_ (22). In endothelial cells, these lipoxins decrease the production of cellular adhesion molecules, inhibit vascular endothelial growth factor (VEGF)-induced migration/proliferation (23), and stimulate prostaglandin I2 (PGI2) and nitric oxide (NO) formation (24). Fibroblasts block matrix metalloproteinase 3 (MMP-3) production (25), and neutrophils block superoxide generation and inhibit peroxynitrite formation (24). All of these effects may account for the decrease in cellular infiltrate and fibrosis induced by 15-epi-LXA_4_ in T. cruzi-infected hearts (13).

However, when the production of 15-epi-LXA_4_ was inhibited, the effect of simvastatin was not completely reversed, suggesting that there may be another component mediating the action of simvastatin. This intermediate may be the Notch pathway. Indeed, in the present model, simvastatin increased Notch activity beyond that induced by the infection itself. Moreover, inhibition of Psn1 activity reversed the effect of simvastatin on not only Notch pathway activity but also inflammation (cardiac cell infiltration, fibrosis, and cytokine production) and heart function, especially the ejection fraction and the systolic diameter of the LV. Thus, these results suggest a causal relationship between the effect of simvastatin and the activity of the γ-secretase involving the Notch pathway in the action of simvastatin, as previously suggested (15). Interestingly, the fibrosis observed after Notch 1 pathway inhibition was similar to that observed with inhibition of 15-epi-LXA_4_ production, suggesting a compound effect of simvastatin involving both Notch 1 and 15-epi-LXA_4_. Reports associating the activity of 15-epi-LXA_4_ with Notch 1 are scarce; however, a recent study demonstrated that lipoxin A_4_ can decrease the activity of this pathway in microglia (26). Thus, in the context of inflammation, these eicosanoids might have a role. Regardless of the case, as the activity of 15-epi-LXA_4_ is not affected by the presence of DAPT, it follows that the effect of this lipid mediator does not involve Notch signaling.

Functional studies in different species or cellular lineages or systems have shown that Notch signaling participates in the control of angiogenic growth, endothelial cell proliferation, and differentiation of arteries and veins (27). Isolectin B4 is considered a marker of endothelial cells. This indicator, together with Ki67, decreased in infected tissues, in agreement with the report that T. cruzi decreases angiogenesis (28). Interestingly, simvastatin also increased the number of cells showing colocalization of isolectin B4 with NICD, a phenomenon that decreases when DAPT is present. Thus, it is probable that the increase in endothelial cells observed with simvastatin might be mediated by Notch activation, which might be part of an angiogenic effect participating in the improvement of the cardiac function observed in this model. Some reports have addressed the notion that Notch may have a functional cardioprotective role during hypoxia/reoxygenation injury (8, 9) although this is still controversial. Moreover, the activation of Notch signaling by artificial means could promote myocardial survival and angiogenesis and improve cardiac function following ischemic injury (8, 9). Indeed, hypoxia can activate several pathways that increase Notch activity, including HIF-2α (29). As with Toll-like receptors (TLRs), the Notch-HIF relationship is reciprocal. Under cardiac ischemia, HIF may activate NICD nuclear translocation (30), thus providing a means to increase angiogenesis to preserve cardiac function, preventing cardiomyocyte loss and fibrosis (6).

There are no previous studies linking statins and 15-epi-LXA_4_ to Notch 1 in mice chronically infected with T. cruzi. However, the impact on cardiac function with simvastatin and eventually its link with the Notch pathway may help elucidate the action of statins on inflamed cardiac tissue. The prevention of chronic cardiac damage in Chagas disease is a therapeutic challenge because regeneration of the fibrotic myocardium is not possible; thus, the present work provides preclinical evidence supporting the use of statins as adjuvants in the treatment of this condition.

## MATERIALS AND METHODS

### Animals.

Eight-week-old BALB/c female mice kept at the Experimental Station of the Molecular and Clinical Pharmacology Program, Institute of Biomedical Sciences (ICBM), Faculty of Medicine, were handled and cared for according to the *Guide for the Care and Use of Laboratory Animals* (31) with food and water *ad libitum*. The approved CBA 0937 FMUCH protocol, from the Bioethics Committee of the Faculty of Medicine, University of Chile, authorized mouse handling and experimental procedures for the present study.

### Parasites.

Dm28c trypomastigotes were harvested from parasites emerging from T. cruzi-infected Vero cell cultures. Then, 1 × 10^5^ parasites were injected intraperitoneally (i.p.) into donor mice. At the peak of parasitemia (usually on the 10th day), cardiac blood was collected and pooled from several donors, and the parasites were counted by a thick blood smear under light microscopic examination. Finally, the trypomastigotes were suspended in sterile saline, and a suspension of 1 × 10^3^ trypomastigotes in 100 μl was injected into each experimental animal. The quality of the infection was determined by measuring parasitemia every other day from tail tip blood samples from 2 days postinfection (dpi) until the parasite was undetectable (32).

### Treatment protocols.

Infected BALB/c mice were treated with 1 mg/kg/day simvastatin (MSD, United Kingdom), 25 μg/kg/day 15-epi-LXA_4_ (Cayman Chemicals, USA), 1 mg/kg/day simvastatin plus 10 mg/kg/day *N*‐[*N*‐(3,5‐difluorophenacetyl)‐l‐alanyl]‐*S*‐phenylglycine *t*‐butyl ester (DAPT; Tocris, United Kingdom), 1 mg/kg/day simvastatin plus 1 mg/kg/day AA861 (a 5-LOX inhibitor; Santa Cruz, USA), and 25 μg/kg/day 15-epi-LXA_4_ plus 10 mg/kg/day DAPT. Simvastatin was dissolved in a suspension of 0.5% carboxymethylcellulose and administered orally once a day, while 15-epi-LXA_4_, DAPT, and AA861 were dissolved in phosphate-buffered saline (PBS) and administered i.p. once a day. The treatments were administered at the chronic phase, from day 60 to day 80 postinfection (p.i.) (13, 33). Finally, heart tissue was obtained after euthanasia with a mixture of 100 mg/kg ketamine and 10 mg/kg xylazine on day 80 p.i.

### Histology, immunohistochemistry, and immunofluorescence staining.

Eighty-day-old hearts from the euthanized mice were fixed in 4% formaldehyde (pH 7.3) for 12 h, dehydrated in alcohol, clarified in xylene, and embedded in paraffin to be sectioned at 5 μm. The paraffin sections were stained with hematoxylin-eosin to observe the architecture and cardiac organization. Additionally, they were stained with Picrosirius red (Sigma-Aldrich, USA) to observe the collagen organization. The images were obtained with a Motic BA310 microscope coupled with a 5.0 MP Moticam camera and analyzed with ImageJ software, version 1.6.

The proteins of the Notch 1 pathway were evaluated in cardiac tissues using the immunoperoxidase technique with Notch 1 (1: 200), NICD (1: 100), and Hes 1 (1: 200) primary antibodies (Abcam, United Kingdom). Staining was performed using a peroxidase and diaminobenzidine kit with a chromophore according to the manufacturer’s instructions (RTU-Vectastain kit; Vector Laboratories, USA). The heart tissue was additionally stained with hematoxylin. The images were obtained with a Motic BA310 microscope coupled with a 5.0 MP Moticam camera and analyzed with ImageJ software, version 1.6. (isotype controls are presented in Fig. S1 in the supplemental material).

For analysis of angiogenesis, the cardiac tissues were evaluated by immunostaining for Ki67 (1:200; Cell Signaling, USA), a cell proliferation marker, conjugated with Alexa 647 (1:200; Invitrogen, USA), isolectin B conjugated with Alexa 594 (1:200; Invitrogen, USA) to identify endothelial cells, and 4′,6′-diamidino-2-phenylindole (DAPI) as nuclear marker. The images were obtained with an Ultra View RS Spinning Disk microscope (Perkin Elmer) with a 40× oil objective (numerical aperture [NA], 1.3). Images were processed from five fields per heart using Fiji software.

### ELISA.

Heart blood from BALB/c mice was collected by intracardiac puncture on day 80 postinfection and centrifuged, and the resultant serum was stored at −80°C. Serum samples were used for TNF-α, IL-1β, and IL-10 analyses. Quantification of cytokines was performed by enzyme-linked immunosorbent assays (ELISAs) using specific mouse monoclonal antibodies from an ELISA Max Deluxe Set for mouse TNF-α, IL-1β, and IL-10 (BioLegend, USA) according to the manufacturer’s instructions. ELISA plates were read at 450 nm in an Asys Expert Plus microplate reader (Biochrome, United Kingdom). All samples were assayed in duplicate.

### Protein extraction and Western blotting.

The paraffin-embedded hearts were deparaffinized by incubation at room temperature in xylene and rehydrated with a graded series of ethanol. The rehydrated heart tissues were homogenized in 20 mM Tris-HCl buffer at pH 9 with 2% (wt/vol) SDS containing a protease inhibitor cocktail (Complete Mini; (Roche). The samples were incubated at 80°C in a dry bath for 2 h. The homogenate was centrifuged at 15,000 × *g* for 15 min at 4°C to remove debris. The protein concentration was measured with a Qubit 4 Fluorometer (Thermo Fisher, USA). Seventy-five micrograms of protein was separated in a 10% sodium dodecyl sulfate-polyacrylamide gel, blotted onto a nitrocellulose membrane, and blocked with 5% bovine serum albumin (BSA) in Tris-buffered saline (TBS)-Tween for 1 h at room temperature. For Ki67, PAGE was performed on a 4% to 20% polyacrylamide gel (Bio-Rad, USA). Membranes were probed with primary antibodies against Notch 1 (1:200; Abcam, United Kingdom), NICD (1:200; Abcam), Hes 1 (1:200; Abcam), Ki67 (1:100; Cell Signaling, USA), and glyceraldehyde-3-phosphate dehydrogenase (GAPDH) (1:100; Santa Cruz Technologies, USA) as loading controls overnight at 4°C and then with peroxidase-conjugated secondary anti-rabbit antibody for 2 h at room temperature. Immunoreactive proteins were detected using enhanced chemiluminescence Immobilon reagent (Merck, Germany) according to the manufacturer’s instructions (Fig. S3).

### RT-qPCR assay.

For evaluation of tissue parasitism, T. cruzi DNA was quantified in the heart samples by quantitative PCR (qPCR) analysis. DNA was isolated using a Wizard Genomic DNA purification kit (Promega, USA) according to the manufacturer’s instructions. DNA was quantified as a function of absorbance at 280 nm using a Varioskan spectrophotometer (Thermo Scientific, USA). qPCR analyses and DNA amplification were performed with SYBR green PCR Master Mix (Bioline, USA) using satellite DNA for T. cruzi Dm28c on an Applied Biosystems 7300 reverse transcription-PCR (RT-PCR) system (Thermo Fisher) using the following primers: forward, 5′-GCTCTTGCCCACAMGGGTGC-3′; reverse, 3′-CAAGCAGCGGATAGTTCAGG-5′. The parasitic load of T. cruzi in the cardiac tissues was calculated from a standard curve constructed using homogenized heart tissues enriched with 10^6^ trypomastigotes of T. cruzi and serially diluted to provide a 7-log curve in a range of 10^−1^ to 10^5^ parasite equivalents (par eq)/ml according to Duffy et al. (34). The limit of detection of the assay was 0.7 par eq/ml ( 95% confidence interval [CI], 0.0089 to 0.7925 par eq/ml) with a limit of quantification of 1.4 par eq/ml of homogenate (Fig. S2).

### Transthoracic echocardiography.

Transthoracic echocardiography was performed using a Vivid-i echocardiograph (General Electric, USA) with a 13 MHz iRL-RS transducer. T. cruzi-infected mice were lightly sedated with isoflurane (1.5%), and the chest was shaved with depilatory cream to facilitate transducer use. The images were obtained in mode B (two dimensions of the different cameras). Echocardiography was obtained at 40 and 80 dpi.

### Statistical analysis.

The results are expressed as the means ± standard errors of the means (SEM) of five or more independent experiments. The statistical analysis was performed with one-way analysis of variance (ANOVA), followed by Dunnett’s *post hoc* test. A *P* value of *≤*0.05 was considered significant. The survival of the mice in the different groups was analyzed by a nonparametric Kaplan-Meier test. All statistical analyses were carried out with Prism, version 6.0, software (GraphPad Prism, USA).

## Supplementary Material

Supplemental file 1
