# Supplementary material for: Simvastatin Improves Cardiac Function through Notch 1 Activation in BALB/c Mice with Chronic Chagas Cardiomyopathy
Source: Antimicrob Agents Chemother. 2020 Jul 22;64(8):e02141-19. doi: 10.1128/AAC.02141-19 (PMC7526820; doi:10.1128/AAC.02141-19)
Supplement: Supplemental file 1 [file AAC.02141-19-s0001.pdf]

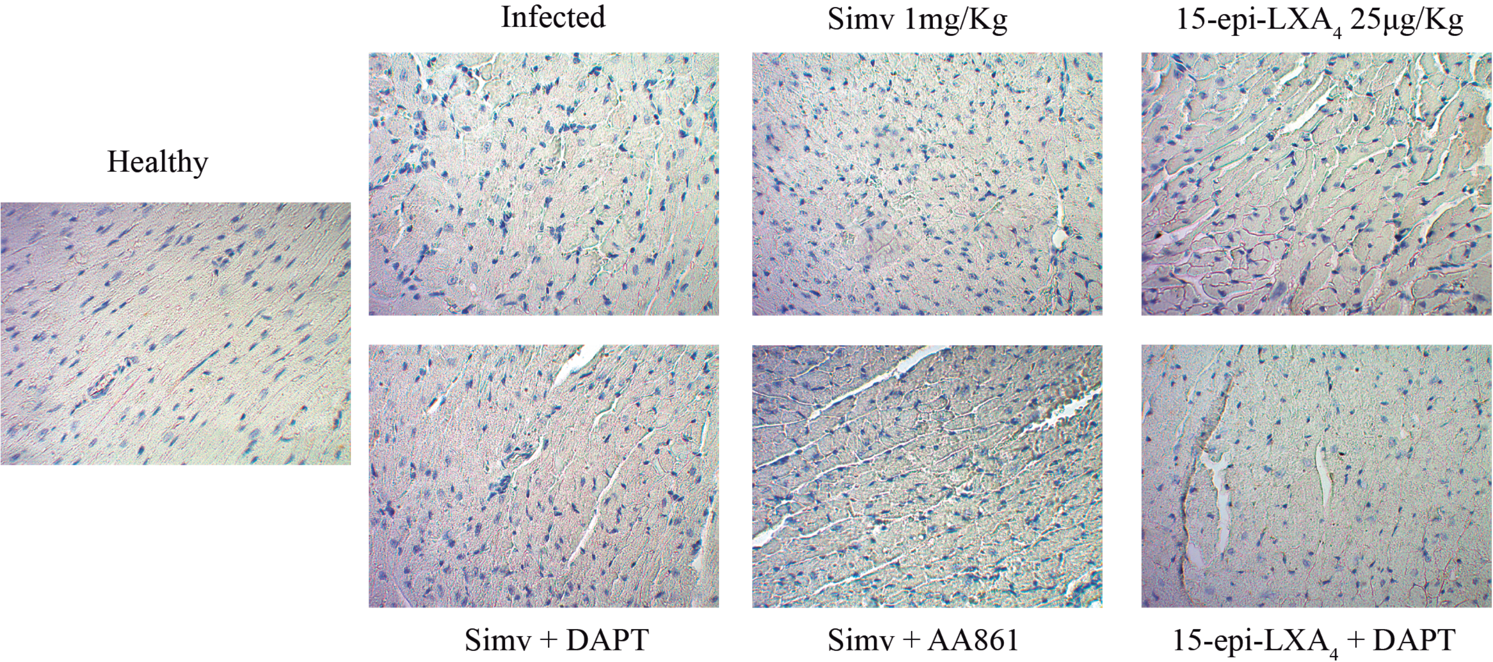

Supplementary Figure 1. Representative isotype control for antibodies used in immunohistochemistry in BALB/c mice cardiac tissues

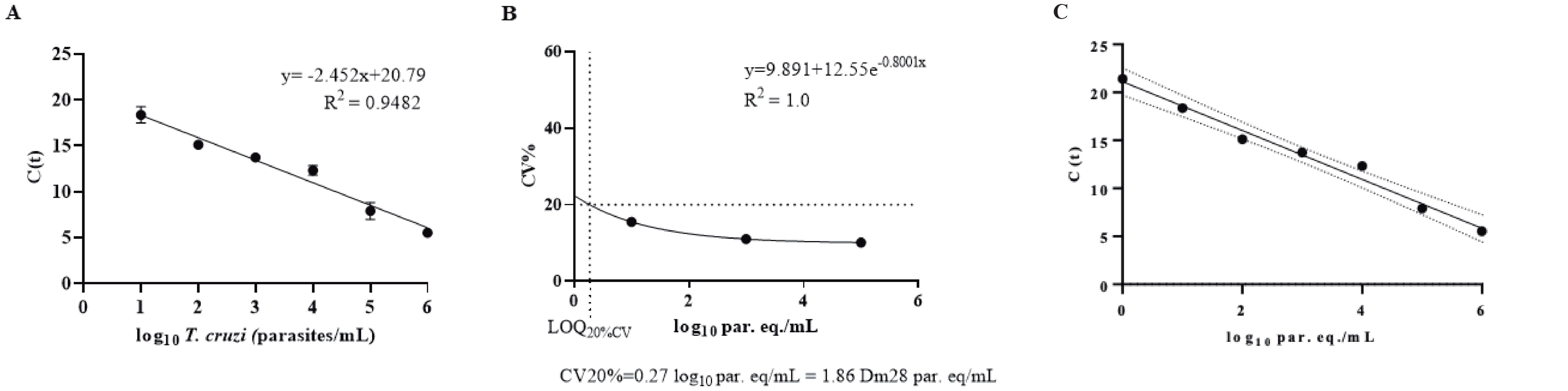

Supplementary Figure 2. A) Dynamic range of *T. cruzi* satellite DNA based qPCR. Results are expressed as the number of parasites/mL of five independent determinations. The dynamic range of qPCR performed with samples reconstituted with Dm28 was 1–10^6 p/mL. B) The LOQ was derived from a 20% threshold value for the coefficient of variation (CV) of measurements of DNA from mice heart homogenates spiked with Dm28 trypomastigotes. Linear least-squares curve fit for the relationship between CV and parasite concentration (log10 par. eq./mL) using GraphPad Prism 8. The derivation of LOQ20%CV is illustrated by dotted lines. C) Limit of detection, obtained by linear regression. LOD 0.026 (CI 95% (0,0089 - 0,7925)) $p < 0.001$

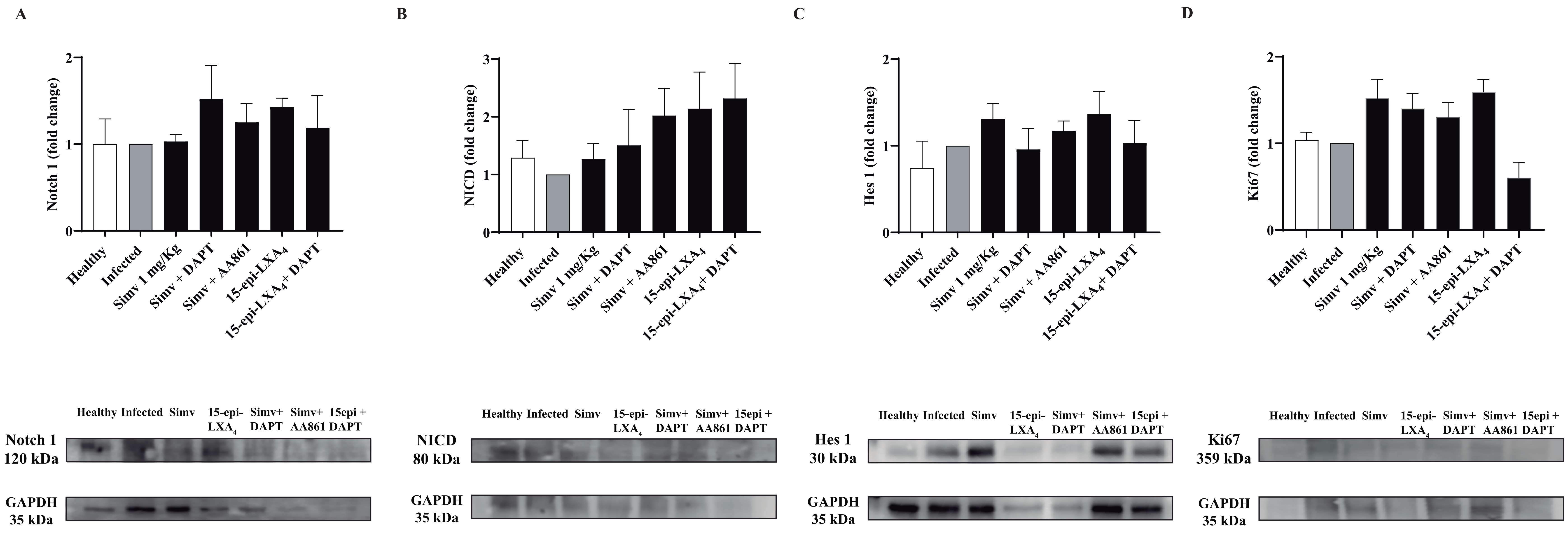

Supplementary Figure 3. BALB/c mice were infected with T. cruzi trypomastigotes and treated for 20 days from 60 to 80 days postinfection with 1 mg/Kg/day simvastatin, 25 µg/kg/day 15-epi-LXA4, simvastatin 1 mg/Kg/day + 10 mg/Kg/day N-[N-(3,5-Difluorophenacetyl)-L-alanyl]-S-phenylglycine t-butyl ester (DAPT) 1 mg/Kg/day simvastatin + 1 mg/Kg/ day AA861, and 15-epi-LXA4 25 µg/Kg/day + DAPT 10 mg/Kg/day. Heart tissues obtained at the 80th day and included in paraffin were xylol deparaffined and rehydrated with ethanol. Hearts were minced and TRIS-PBS lysed with protease inhibitors. Total protein changes were evaluated by western blot as a relative quantification using GAPDH as housekeeping for A) Notch 1, B) NICD, C) Hes 1 and D) Ki67 (4-20% SDS-PAGE). The bottom panel is a representative image for every blot. The data are expressed as mean ± SEM of five independent determinations. One-way ANOVA analysis was performed.
